# Supplementary material for: The probability of reducing hospitalization rates for bronchiolitis with epinephrine and dexamethasone: A Bayesian analysis
Source: PLoS One. 2025 May 16;20(5):e0318853. doi: 10.1371/journal.pone.0318853 (PMC12083810; doi:10.1371/journal.pone.0318853)
Supplement: S1 File — (DOCX) [file pone.0318853.s001.docx]

**Supplementary Material**

***Supplementary Material A***

| Study | Treatment | Reference | Outcome studied | RR | Sample size |
| --- | --- | --- | --- | --- | --- |
| S. Schuh et al. (2001) (1) | Dex + Salbutamol | Salbutamol | Admission to hospital within 240 minutes after initial treatment | 0.44 | 70 |
| H. Corneli et al. (2007) (2) | Dex | Placebo | Admission to hospital within 240 minutes after initial treatment | 0.97 | 600 |
| M. Mesquita et al. (2009) (3) | Dex + Epi | Epi | Admission to hospital within 240 minutes after initial treatment | 1.11 | 65 |
| S. Hariprakash et al. (2003) (4) | Epi | Placebo | Admittance to hospital | 0.80 | 75 |
| Barlas et al. (1998) (5) | Epi | Placebo | Admission to hospital | 0.12 | 30 |
| Goebel et al. (2000) (6) | Prednisone + albuterol | Albuterol | Hospitalized later in the study | 0.59 | 32 |

*Table A.1: Summary table of past studies used in the design of reference and data-driven priors in the Bayesian analysis.*

| 1. | Schuh S, Babl FE, Dalziel SR, et al. Practice variation in acute bronchiolitis: a pediatric emergency research networks study. *Pediatrics*. 2017; 140(6): e20170842. |
| --- | --- |
| 2. | Corneli H, Zorc J, Mahajan P, et al. A multicenter, randomized, controlled trial of dexamethasone for bronchiolitis. *New England Journal of Medicine*. 2007; 357(4): 331-339. |
| 3. | Mesquita M, Castro-Rodríguez J, Heinichen L, Fariña E, Iramain R. Single oral dose of dexamethasone in outpatients with bronchiolitis: a placebo controlled trial. *Allergologia et immunopathologia*. 2009; 37(2): 63-67. |
| 4. | Hariprakash S, Alexander J, Carroll W, et al. Randomized controlled trial of nebulized adrenaline in acute bronchiolitis. *Pediatric allergy and immunology*. 2003; 14(2): 134-9. |
| 5. | Barlas C, et al. Racemic adrenaline and other treatment regimens in mild and moderate bronchiolitis. *Cocuk Sagligi Ve Hastalikari Dergisi*; 1998: 41(2): 155-166. |
| 6. | Goebel J, Estrada B, Quinonez J, Nagji N, Sanford D, Boerth RC.  Prednisolone plus albuterol versus albuterol alone in mild to moderate bronchiolitis. *Clinical pediatrics*. 2000; 39(4): 213-220. |

***Supplementary Material B***

*Table B.1: Summary table of group 2 (Epi) reference prior characteristics: median RR, standard deviation (SD) of log-RR, probability of RR smaller than various thresholds and rationale behind each probability distribution.*

| Prior Belief | Median Relative Risk (RR) | SD of log-RR | Equivalent Prior Sample Size | Probability RR is below threshold (in %) | | | |
| --- | --- | --- | --- | --- | --- | --- | --- |
|  |  |  |  | 1 | 0.9 | 0.8 | 0.6 |
| **Reference Priors** | | | | | | | |
| Minimally Informative | 1 | 10 | ~0 | 50 | 50 | 49 | 48 |
| Strongly Enthusiastic | 0.80 | 0.13 | 610 | 95 | 80 | 48 | 1 |
| Moderately Enthusiastic | 0.80 | 0.13 | 598 | 95 | 80 | 48 | 1 |
| Moderately Skeptical | 1 | 0.13 | 599 | 50 | 21 | 5 | 0 |
| Strongly Skeptical | 1 | 0.13 | 573 | 50 | 21 | 5 | 0 |
| **Data-Driven Priors** | | | | | | | |
| 100% Weighting | 0.74 | 0.21 | 223 | 92 | 82 | 64 | 16 |
| 50% Weighting | 0.74 | 0.30 | 116 | 84 | 74 | 60 | 24 |
| 10% Weighting | 0.74 | 0.67 | 25 | 67 | 61 | 55 | 38 |

*Table B.2: Summary table of group 3 (Dex) reference prior characteristics: median RR, standard deviation (SD) of log-RR, probability of RR smaller than various thresholds and rationale behind each probability distribution.*

| Prior Belief | Median Relative Risk (RR) | Standard Deviation of log-RR | Equivalent Prior Sample Size | Probability RR is below threshold | | | |
| --- | --- | --- | --- | --- | --- | --- | --- |
|  |  |  |  | 1 | 0.9 | 0.8 | 0.6 |
| **Reference Priors** | | | | | | | |
| Minimally Informative | 1 | 10 | ~0 | 50 | 50 | 49 | 48 |
| Strongly Enthusiastic | 0.93 | 0.21 | 195 | 63 | 44 | 24 | 2 |
| Moderately Enthusiastic | 0.93 | 0.21 | 192 | 63 | 44 | 24 | 2 |
| Moderately Skeptical | 1 | 0.21 | 209 | 50 | 31 | 15 | 1 |
| Strongly Skeptical | 1 | 0.21 | 195 | 50 | 31 | 15 | 1 |
| **Data-Driven Priors** | | | | | | | |
| 100% Weighting | 0.97 | 0.10 | 973 | 62 | 57 | 3 | 0 |
| 50% Weighting | 0.97 | 0.14 | 536 | 59 | 30 | 9 | 0 |
| 10% Weighting | 0.97 | 0.32 | 114 | 54 | 41 | 27 | 6 |

***Supplementary Material C***

|  | Posterior median  (95% Credible Interval) | Posterior probability of RR < threshold (in %) | | | |
| --- | --- | --- | --- | --- | --- |
|  |  | RR < 1 | RR < 0.9 | RR < 0.8 | RR < 0.6 |
| **Reference Priors** | | | | | |
| Minimally informative | 0.90 (0.64, 1.26) | 72 | 49 | 23 | 1 |
| Strongly enthusiastic | 0.84 (0.68, 1.02) | 96 | 76 | 33 | 0 |
| Moderately enthusiastic | 0.86 (0.70, 1.04) | 94 | 69 | 25 | 0 |
| Skeptical | 0.98 (0.80, 1.19) | 59 | 21 | 3 | 0 |
| Strongly Skeptical | 1.00 (0.82, 1.22) | 50 | 15 | 1 | 0 |
| **Data-Driven Priors** | | | | | |
| 100% weighting | 0.87 (0.68, 1.11) | 87 | 61 | 26 | 0 |
| 50% weighting | 0.89 (0.67, 1.17) | 80 | 54 | 23 | 0 |
| 10% weighting | 0.90 (0.66, 1.24) | 73 | 49 | 22 | 1 |

*Table C.1: Summary table of group 2 (Epi) reference posterior characteristics: median RR, 95% posterior credible interval-RR, probability of RR smaller than various thresholds.*

|  | Posterior median  (95% Credible Interval) | Posterior probability of RR < threshold (in %) | | | |
| --- | --- | --- | --- | --- | --- |
|  |  | RR < 1 | RR < 0.9 | RR < 0.8 | RR < 0.6 |
| **Reference Priors** | | | | | |
| Minimally informative | 0.98 (0.71, 1.36) | 54 | 30 | 11 | 0 |
| Strongly enthusiastic | 0.94 (0.74, 1.19) | 70 | 37 | 10 | 0 |
| Moderately enthusiastic | 0.97 (0.76, 1.23) | 60 | 27 | 6 | 0 |
| Skeptical | 1.03 (0.80, 1.31) | 42 | 15 | 2 | 0 |
| Strongly Skeptical | 1.06 (0.83, 1.35) | 31 | 9 | 1 | 0 |
| **Data-Driven Priors** | | | | | |
| 100% weighting | 0.98 (0.83, 1.15) | 59 | 15 | 1 | 0 |
| 50% weighting | 0.98 (0.80, 1.21) | 56 | 20 | 2 | 0 |
| 10% weighting | 0.98 (0.74, 1.31) | 54 | 27 | 8 | 0 |

*Table C.2: Summary table of group 3 (Dex) reference posterior characteristics: median RR, 95% posterior credible interval-RR, probability of RR smaller than various thresholds.*

***Supplementary Material D***


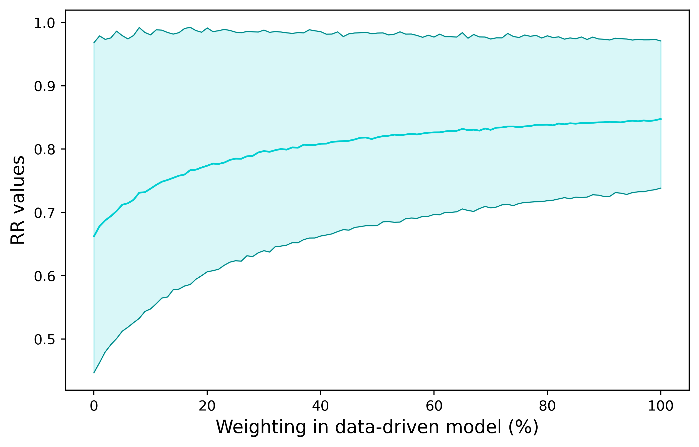

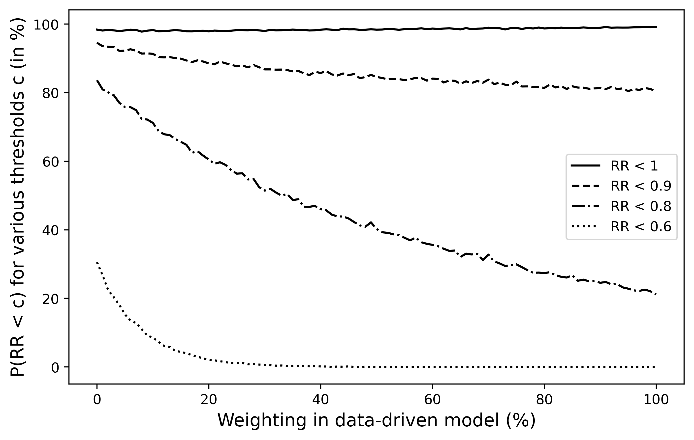


*Figure D.1: Posterior distribution characteristics for the epinephrine-dexamethasone treatment arm relative risk: posterior median, equi-tailed 95% credible interval and posterior probabilities of RR being smaller than various thresholds.*

We computed posterior distributions using data-driven priors with increasing weights between 0% and 100% (see Figure D.1). Overall, the RR median increases with a higher weighting for data-driven priors, indicating that the probability of having a reduction in hospitalizations increases with more emphasis on the CanBEST data. The left-hand side of Figure D.1 displays the median RR and 95% credible intervals as the weighting in the data-driven priors increases. The median increases, reaching 0.85 with approximately 60% prior weight. The 95% credible intervals also become smaller as the weighting of the prior increases, as there is more data available to estimate the RR. The right-hand side of Figure D.1 displays the probability that the RR is below the thresholds 1, 0.9, 0.8, and 0.6. The probability of a reduction in hospitalization (RR < 1) remains close to 100% irrespective of the prior weights, indicating a high chance that EpiDex reduces hospitalizations based on all the currently available evidence.

**Figure D.1 legend (left image)**:

None

**Figure D.1 legend (right image)**:

- Solid line: RR < 1
- Dashed line: RR < 0.9
- Dash-dotted line: RR < 0.8
- Dotted line: RR < 0.6
